# Changes in Circulating Adipokine Levels in COVID-19 Patients

**DOI:** 10.3390/jcm13164784

**Published:** 2024-08-14

**Authors:** Tomasz Wikar, Mateusz Rubinkiewicz, Dominika Stygar, Elżbieta Chełmecka, Urszula Popiela, Wysocki Michał, Piotr Tylec, Barbara Maziarz, Michał Kukla

**Affiliations:** 1 2nd Department of General Surgery, Jagiellonian University Medical College, 31-066 Kraków, Poland; 2 Department of Medical Education, Jagiellonian University Medical College, 31-066 Krakow, Poland; 3 Department of Physiology, Faculty of Medical Sciences in Zabrze, Medical University of Silesia, 41-808 Zabrze, Poland; 4 Department of Medical Statistic, Faculty of Pharmaceutical Sciences in Sosnowiec, Medical University of Silesia, 40-055 Sosnowiec, Poland; 5 Department of General Surgery and Surgical Oncology, Ludwik Rydygier Memorial Hospital, 31-826 Kraków, Poland; 6 Faculty of Medicine, Jagiellonian University Medical College, 31-066 Kraków, Poland; 7 Department of Diagnostics, University Hospital, 30-688 Kraków, Poland; 8 Department of Internal Medicine and Geriatrics, Faculty of Medicine, Jagiellonian University Medical College, 31-066 Krakow, Poland; 9 Department of Endoscopy, University Hospital in Kraków, 30-688 Krakow, Poland

**Keywords:** adipokine, coronavirus, COVID-19, leptin, obesity, SARS-CoV-2, visfatin

## Abstract

**Objective**: The COVID-19 pandemic has posed significant global health challenges. Despite extensive research efforts, the inflammatory response triggered by SARS-CoV-2 remains to be further explored and understood. Our study aims to examine the changes in serum concentrations of pro-inflammatory adipokines—visfatin and leptin—in COVID-19 patients in relation to a healthy control group. **Patients/Materials/Subjects and Methods**: The study consisted of forty COVID-19 patients and twenty-four healthy patients in the control group. Two serum samples were collected: upon admission and on the seventh day of hospitalization. Concentrations of visfatin and leptin in the serum, alongside routine biochemical parameters, were measured using enzyme immunoassay or enzyme-linked immunosorbent assay kits. The Shapiro–Wilk test was used to assess normality. Differences between independent groups were compared using the Mann–Whitney U test and Kruskal–Wallis ANOVA. Correlations were evaluated with Spearman’s rank correlation coefficient. **Results**: Our findings revealed significantly lower visfatin levels in COVID-19 patients compared to the control group upon admission (4.29 ng/mL, (3.0–6.88 ng/mL) vs. 37.16 ng/mL (24.74–50.12 ng/mL), *p* < 0.001 for visfatin 1 and 52.05 ng/mL, (31.2–69.66 ng/mL) vs. 37.16 ng/mL (24.74–50.12 ng/mL), *p* = 0.048 for visfatin 2). The visfatin level of COVID-19 patients returned to the normal levels, established in the control group. However, there was no significant difference in leptin levels between the two groups (*p* = 0.270 for leptin 1 and *p* = 0.129 for leptin 2). There was a positive correlation between BMI and leptin concentration (r = 0.66 and *p* = 0.00). Moreover, it was discovered that COVID-19 independently reduces visfatin levels during the first day of illness. **Conclusions**: The results of our research suggest that the onset of COVID-19 infection is correlated to visfatin levels. Association with leptin levels remains inconclusive. Further research is imperative to elucidate the intricate role of visfatin and leptin in SARS-CoV-2 infection and their potential as biomarkers for COVID-19 severity and prognosis.

## 1. Introduction

COVID-19 is caused by severe acute respiratory syndrome coronavirus-2 (SARS-CoV-2), an enveloped, single-stranded, positive-sense RNA virus that originates from the Coronaviridae family. The course of the disease may be asymptomatic, but manifestations include a variety of gastrointestinal, cardiovascular, and respiratory symptoms [1]. Among those listed, pulmonary dysfunction is the most common cause of death. Global concerns were raised after 11 March 2020, when the WHO declared a pandemic due to a spike in new COVID-19 cases and deaths [2]. As of today, over 6.9 million deaths have been reported, ranking SARS-CoV-2 virus as the seventh most lethal virus in history.

A global determination to understand the disease has been put forward, resulting in more than 334 thousand publications in PubMed. Despite such efforts, the inflammatory response is not yet fully understood. Nevertheless, it is evident that the immune system is involved in battling the virus itself, and its dysregulation leads to concomitant damage to infected tissues. It is known that the innate immune system’s pathogen recognition receptors (PRRs) identify RNA from the virus and activate the inflammatory production of cytokines like interleukin-1 and -6, tumor necrosis factor-alpha, and interferons I and III. The adaptive immune response is triggered when B and T lymphocyte receptors bind with viral antigens. T cells can act through CD4+ cells, helping in the development of immune response and tissue repair, or through CD8+ T cells that kill infected cells. B cells produce antibodies that attempt to counteract the virulent potential of the pathogen [3,4,5]. Dysregulation in immune response leads to cytokine release syndrome (CRS). Scientific data imply that patients with comorbidities, particularly obesity, defined as body mass index (BMI) ≥ 30 kg/m^2^, are at a greater risk of severe course of the disease and lethal complications [6,7,8].

The dysregulation of the immune response in obese individuals is recognized as a significant factor contributing to the increased severity of pulmonary infections. Obesity is associated with a higher number and heightened inflammatory capacity of monocytes, macrophages, neutrophils, and CD4 T cells. Notably, the pro-inflammatory state of neutrophils and macrophages in obese patients leads to a substantial increase in cytokine expression and elevated levels of reactive oxygen species [9]. Obesity is linked to adipose tissue dysfunction, which not only predisposes individuals to metabolic complications but also significantly contributes to chronic low-grade systemic inflammation, altered immune cell composition, and impaired immune function. This dysfunction impacts the susceptibility to and outcomes of viral diseases, as obese individuals are more prone to developing infections and experience delayed recovery compared to their normal-weight counterparts [10].

Furthermore, studies have shown that adverse course of COVID-19 in Caucasian adults is strongly correlated with a higher content of visceral fat (VF) [11].

Briefly, VF is responsible for the secretion of adipokines, which are biologically active molecules with pro- and anti-inflammatory effects influencing immune response [12]. We decided to investigate serum concentrations of two pro-inflammatory adipokines: visfatin and leptin. The serum levels of visfatin and leptin in COVID-19 patients have been found to be significantly higher than in healthy control groups. However, specific numerical values and statistical data vary across studies [13,14,15]. Visfatin, also known as extracellular nicotinamide phosphoribosyltransferase (eNAMPT) or pre-B cell colony-enhancing factor 1 (PBEF-1), has various biological roles and is produced by different types of cells, including lymphocytes, neutrophils, monocytes, adipocytes, hepatocytes, and pneumocytes. Higher levels of visfatin are commonly associated with both acute and persistent inflammatory conditions. Initially identified as PBEF for its role in supporting the development of B cell precursors alongside IL-7 and stem cell factor, visfatin is also recognized as an immunomodulating adipokine with pro-inflammatory properties. Visfatin helps with B cell maturation, prevents neutrophil apoptosis, enhances leukocyte activation, and increases the production of adhesion molecules and pro-inflammatory cytokines [16]. Leptin, in general, enhances the immune response by activating antigen-presenting cells (APCs), supporting the function and proliferation of Th1 cells, and mediating the secretion of pro-inflammatory cytokines such as TNF-α, IL-2, or IL-6 [17]. Our analysis aimed to assess the changes in visfatin and leptin serum concentrations among COVID-19 patients upon admission and on the seventh day of the disease (t2) compared to a healthy control group.

## 2. Materials and Methods

A total of 40 COVID-19 patients, consisting of 20 females and 20 males, were included in the study, of which 20 had hypertension, 11 had obesity of first degree, and 8 had type 2 diabetes. Patients complained mainly of dyspnea (12), cough (11), and dysosmia (3), and 11 of them had radiological features of pneumonia. 

The study was conducted in compliance with the Declaration of Helsinki (2000) of the World Medical Association and was approved by the Ethics Committee of the Jagiellonian University in Cracow (resolution number 1072.6120.157.2020). Informed consent was waived as the analysis was performed using residual plasma samples collected for routine laboratory tests upon admission (t1) and on the seventh day of hospitalization (t2). Patients were hospitalized due to positive RT PCR SARS-CoV-2 test in combination with the symptomatic course of the disease or the inability to isolate at home between November of 2020 and February of 2021. In this timeframe, the most common SARS-CoV-2 variant was VOC 202012/01 [18].

Patients were eligible for inclusion based on a positive reverse transcriptase polymerase chain reaction (RT-PCR) assay for SARS-CoV-2 from a nasal and/or throat swab. Information on existing and past co-morbidities and current medication use was extracted from electronic medical records. Patients were excluded if they had hepatitis B virus (HBV) or hepatitis C virus (HCV) infections; human immunodeficiency virus (HIV) co-infection; history of drug abuse; presence of neoplastic, thyroid disease, chronic renal failure, mental illnesses, chronic liver disease, or cirrhosis due to primary sclerosing cholangitis (PSC); primary biliary cholangitis (PBC); autoimmune hepatitis (AIH); or alcohol cirrhosis (AC). The control group consisted of 24 patients who were scheduled for cholecystectomy due to cholelithiasis and were otherwise healthy. These patients had no gastrointestinal or chronic liver diseases, history of smoking or alcohol intake, or systemic diseases. Analysis of the patients was stratified based on their BMI (<30 and ≥30 kg/m^2^) and oxygen blood saturation levels.

Serum samples were collected from peripheral blood using centrifugation. The concentrations of visfatin and leptin in the serum were measured in duplicate using commercially available enzyme immunoassay (EIA) or enzyme-linked immunosorbent assay (ELISA) kits: visfatin (NAMPT) Human ELISA (BioVendor—Laboratorni Medicina a.s., Brno, Czech Republic) with a sensitivity of 30 pg/mL, intra-assay error of 5.58%, and inter-assay error of 6.24%; leptin ELISA Kit (BioVendor Laboratorni Medicina a.s., Brno, Czech Republic) with a sensitivity of 0.5 ng/mL, intra-assay error of 7.2%, and inter-assay error of 8%.

Routine methods were employed to measure the remaining biochemical parameters, including full blood count, renal function tests, serum ammonia, and C-reactive protein (CRP). All patients included in this study were treated according to the local treatment protocol, which included therapeutic doses of low-molecular-weight heparin and 6 mg dexamethasone daily. Patients received Remdesivir if the duration of symptoms was less than five days prior to hospitalization. Every patient exhibiting radiological signs of pneumonia and elevated white blood cell count received empiric antibiotic therapy with 1.0 g of ceftriaxone twice a day and 0.4 g of ciprofloxacin twice a day. The course of the illness was mild to moderate. Two of the forty patients were admitted to ICU due to respiratory failure. 

## 3. Statistical Analysis 

Continuous variables were expressed as median with inter-quartile range (IQR). The Shapiro–Wilk test was conducted to assess the distribution. The Mann–Whitney U test and Kruskal–Wallis ANOVA tests were conducted to test for statistical significance of differences in the variables under study for independent groups. Correlations were examined using Spearman’s rank correlation coefficient. Values of *p* < 0.05 were considered to be statistically significant. The statistical analysis was performed using STATISTICA 10.0 (StatSoft Polska Sp z o.o., Cracow, Poland)

## 4. Results 

This study included a total of 64 participants, of which 40 (62.5%) were COVID-19 RT PCR patients and 24 (37.5%) were controls. The median age of the COVID-19 patients and controls was 63 (37–76) and 58 (34–71) years, respectively, with no statistically significant difference (*p* = 0.56). In terms of gender distribution, both groups had similar proportions of males and females, with 50% males and 50% females in the COVID-19 group and 42% males and 58% females in the control group (*p* = 0.518). The waist circumference was also similar between the two groups, with median values of 94.00 (80.00–110.00) cm in the COVID-19 group and 95.50 (81.00–102.50) cm in the control group (*p* = 0.765). The median BMI in the COVID-19 group was 25.69 (22.81–28.71) kg/m^2^, and in the control group, it was 26.74 (24.27–30.31) kg/m^2^, with no statistically significant difference (*p* = 0.230). The temperature was reported only for COVID-19 patients and had a median value of 36.1 (Q1–Q3: 36–36.35). 

The median serum concentrations of visfatin were significantly lower in the COVID-19 patients than in the control group for visfatin 1 [4.29 ng/mL, (3.0–6.88 ng/mL) vs. 37.16 ng/mL (24.74–50.12 ng/mL), *p* < 0.001] and significantly higher for visfatin 2 [52.05 ng/mL, (31.2–69.66 ng/mL) vs. 37.16 ng/mL (24.74–50.12 ng/mL), *p* = 0.048]. The study found an increase in the median levels of visfatin from visfatin 1 to visfatin 2 in COVID-19 patients by 47.83 ng/mL (26.9–62.65 ng/mL) with a *p*-value of <0.001.

The median levels of leptin in COVID-19 patients were 7476.3 pg/mL (Q1–Q3: 2471.6–21,695.15 pg/mL) for leptin 1 and 6543.75 pg/mL (Q1–Q3: 94,302.5–12,064 pg/mL) for leptin 2, while the control group had levels of 12,586.75 pg/mL (Q1–Q3: 4786.33–22,446.51 pg/mL) for leptin 1 and no recorded levels for leptin 2. The difference in leptin levels between the two groups was not statistically significant (*p* = 0.270 for leptin 1 and *p* = 0.129 for leptin 2). The study found a decrease in the median levels of leptin from leptin 1 to leptin 2 in COVID-19 patients by −1530.5 pg/mL (Q1–Q3: −12,121.3; 3524.03) with a *p*-value of 0.076. 

**Figure 1:**
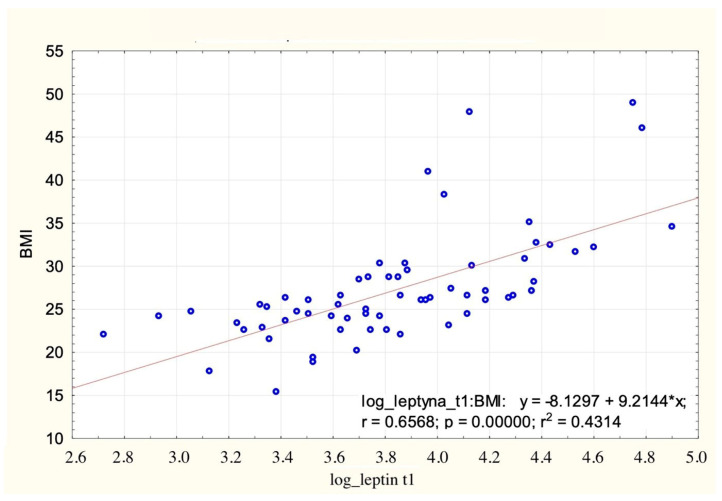
BMI, [kg/m^2^] distribution relative to log_leptin t1.

BMI was positively correlated with serum leptin concentration (r = 0.66), which is presented in Figure 1.

**Figure 2:**
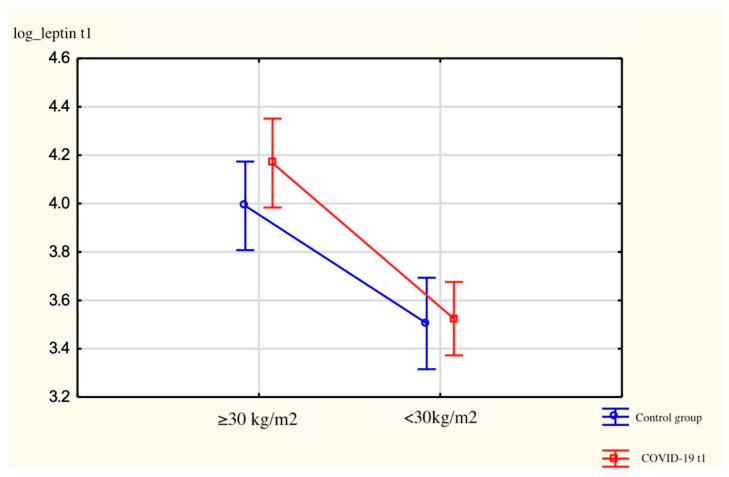
Representing log_leptin t1 serum levels in patients divided in subgroups: ≥30 kg/m^2^, <30 kg/m^2^. Vertical bars indicate a 0.95 confidence interval.

There were no statistically significant differences between BMI (<30 and ≥30 kg/m^2^) groups (*p* = 0.43), as seen in Figure 2.

**Figure 3:**
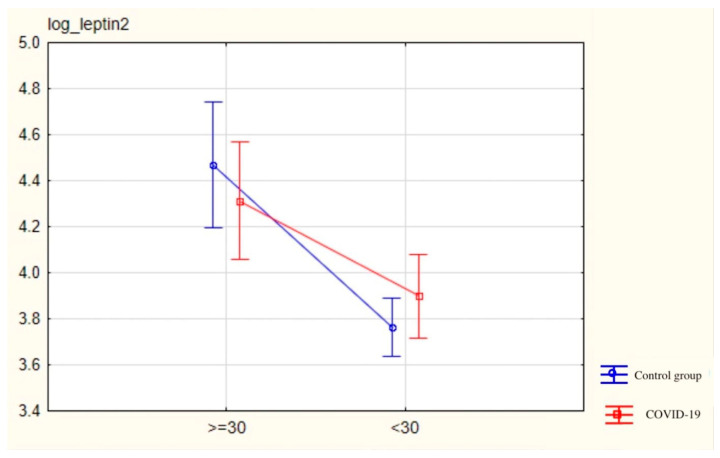
Representing log_leptin t2 serum levels in patients divided into subgroups ≥ 30 kg/m^2^, <30 kg/m^2^. Vertical bars indicate a 0.95 confidence interval.

In subgroups with lower blood oxygenation levels (<95%), primary high GGTP (GGTP activity > 50 U/L), alimentary symptoms [%] (diarrhea—22.5%, vomiting/nausea—25.0%, abdominal pain—2.5%, dysgeusia—5.0%), and respiratory symptoms [%] (cough 25.0%, dyspnea 5.0%, pneumonia 27.5%), we did not find any statistically significant differences in visfatin and leptin serum concentrations, as presented in Figure 3 and Table 1, Table 2, Table 3 and Table 4.

Overall, the results suggest that the beginning of SARS-CoV-2-induced infection is associated with lower serum levels of visfatin and its return to normal on the seventh day of infection but not with significant changes in leptin levels.

## 5. Discussion

Adipokines can have local, peripheral, and central effects [19]. Numerous studies conducted recently pointed out that the connection between obesity and the severity of COVID-19 is becoming increasingly apparent; although, the underlying mechanisms are not fully understood [20,21,22]. On the other hand, the link between obesity and inflammation is well-recognized, with surplus nutrients triggering a metabolic pathway that ultimately activates cytokines, causing a mild inflammatory response. Clinical data show that COVID-19 patients exhibit elevated cytokine levels, termed as “cytokine storm”, and have increased inflammation. This might explain the connection between COVID-19 and obesity. Our study showed an increased level of leptin in patients with higher BMI and no correlation of leptin level with progression of COVID-19 infection. Our results revealed a depletion in visfatin levels on the first day of the disease, followed by a return to control group levels of plasma concentrations on the seventh day. To the best of our knowledge, this is the first time this has been shown in COVID-19 patients.

According to our research, COVID-19 patients exhibited notably lower visfatin plasma concentrations than the control group on day one of hospitalization and higher visfatin levels on day seven of hospitalization compared to healthy individuals. Our study did not find a correlation between the severity of the infection and visfatin levels, unlike Antine W. Flikweert et al., who showed higher visfatin levels in severe and critical COVID-19 patients compared to those with mild cases [13]. Interestingly, in the abovementioned study, visfatin levels were even higher in critical COVID-19 patients compared to those with critical conditions unrelated to COVID-19, indicating a possible specific correlation with severe COVID-19. Another study by Mermutluoglu and Tekin also showed that visfatin levels measured in COVID-19 patients were significantly higher than in the healthy control group [14]. Visfatin is generated by visceral adipose tissue, and its expression is linked to obesity. Its secretion can also be affected by inflammatory cytokines and lipopolysaccharides. Additionally, visfatin has been found to be increased in acute lung inflammation and sepsis and associated with pro-inflammatory responses and endothelial dysfunction. Recent experiments on animals have demonstrated that targeting visfatin with a humanized neutralizing antibody can decrease lung injury and improve respiratory compliance in models of ARDS [23]. Although our study did not find a significant relationship between visfatin levels and clinical variables, we showed using a multivariate regression model that SARS-CoV-2 infection independently lowers visfatin levels on day 1 of hospitalization. We believe that further research is necessary to explore the potential role of visfatin in COVID-19-induced ARDS and whether it can predict disease severity and outcome. Conducting longitudinal studies to investigate circulating visfatin levels in COVID-19 patients could offer valuable insights into its potential as a biomarker.

The correlation between plasma leptin levels and COVID-19 severity has been previously investigated, but the findings have been inconsistent. Some studies reported elevated leptin levels in COVID-19 patients in the ICU [24], while others found a decrease in leptin levels in severe cases compared to mild and moderate ones [13]. Variables such as age, sex, and BMI can affect adipokine levels [25,26,27], including leptin and adiponectin, but not all previous studies considered these factors or used healthy controls for comparison. Leptin is involved in various physiological processes including angiogenesis, hematopoiesis, and both adaptive and innate immunity. Additionally, it affects gastric motility, the olfactory epithelium, and hunger sensation. These systems appear to be impacted in severe COVID-19 infections [28]. However, a recent meta-analysis [29] did not show a significant association between leptin levels in COVID-19 patients compared to control groups. These findings correspond to our work but should be interpreted with caution due to the limited number of studies included in the abovementioned metanalysis and the limited patient count in this work.

In our study, we show a correlation between BMI and leptin serum concentrations, but our data did not show a decrease in leptin levels during infection, as in the paper by Di Filippo et al. [30]. However, other studies observed that dexamethasone treatment increased circulating leptin levels, in agreement with previous experimental studies that showed dexamethasone to be a potent stimulator of leptin production [13]. Therefore, we hypothesize that in our study, a reduction in leptin levels might be overlooked by dexamethasone stimulation. It is believed that systemic adipokines, including leptin, may contribute to endothelial activation and dysfunction in COVID-19 patients with obesity [12]. However, we did not find a direct positive relationship between leptin and IL-6 levels in hospitalized COVID-19 patients. Previous studies have also failed to observe an increase in leptin levels in inflammatory conditions such as sepsis, experimental endotoxemia, and HIV infection despite an elevation in IL-6 levels [19,31,32]. This suggests that leptin may function as an inflammatory mediator in some situations but not in others, such as COVID-19. A recent in vitro study conducted by Ter Ellen BM et al. [33] demonstrated that recombinant leptin and other adipose tissue mediators did not cause an inflammatory response or promote SARS-CoV-2 infection in endothelial cells.

Our study is constrained by a number of limitations. Primarily, the cohort size is comparatively small, limiting the generalizability of the findings. Additionally, the patient sample was predominantly characterized by mild COVID-19 cases, with no individuals requiring intensive care unit (ICU) admission. Consequently, the study did not evaluate changes in leptin and visfatin levels in patients experiencing critical deterioration. Furthermore, the control group was composed of patients with cholelithiasis, and the potential impact of cholelithiasis on adipokine levels was not assessed in this research.

## 6. Conclusions

In conclusion, our research unveiled novel findings that visfatin levels at the beginning of SARS-CoV-2-induced viral infection were significantly lower than those in the control group and, over the seven-day course, went back to the normal levels established in the control group. The shift in median levels of visfatin, observed from the initial to subsequent measurements in COVID-19 subjects, highlights the impact of the virus. Despite showing a trend, leptin levels did not exhibit a significant statistical variance between the two groups. Notably, BMI maintained a positive correlation with serum leptin concentration but did not present substantial differences across varied BMI categories. Delving into subgroups with distinct symptoms, we identified no marked discrepancies. Collectively, our data imply that the commencement of COVID-19 is linked with alterations in visfatin levels, while changes in leptin remain inconclusive. Emphasizing the entirety of our findings, further research is imperative to understand the intricate role of these factors on SARS-CoV-2 infection.

## Figures and Tables

**Table 1:** The baseline characteristics and laboratory data of patients (t1) and healthy volunteers.

	COVID-19	Control Group	*p*-Value
	n = 40 (62.5%)	n = 24 (37.5%)	n/a
Age, years (Q1–Q3)	63 (37–76)	58 (34–71)	0.56
Males/females, n (%)	20/20 (50%/50%)	10/14 (42%/58%)	0.518
Waist circumference, [cm]	94 (80–110)	95.5 (81–102.5)	0.765
BMI, [kg/m^2^]	25.69 (22.81–28.71)	26.74 (24.27–30.31)	0.230
HCT, %	33.00 (29.45–28.8)	41.70 (39.05–44.25)	<0.001
HGB, g/dL	11.30 (9.65–29.45)	14.25 (13.20–15.25)	<0.001
RBC, [10^6^/µL]	3.79 (3.34–4.45)	4.59 (4.40–4.89)	<0.001
WBC, [10^3^/µL]	6.93 (5.14–11.00)	6.59 (5.18–7.92)	0.371
NEU, [10^3^/µL]	5.50 (3.71–7.78)	3.42 (2.83–4.60)	<0.001
LYM, [10^3^/µL]	1.05 (0.77–1.87)	2.11 (1.56–2.99)	<0.001
PLT, [10^3^/µL]	202.50 (139.50–256.00)	259.50 (225.50–296.50)	0.005
CRP, [mg/L]	52.75 (28.85–123.50)	1.28 (0.50–3.70)	<0.001
PCT, [ng/mL]	0.13 (0.02–0.55)	0.02 (0.01–0.04)	0.033
ALP, [IU/L]	64.50 (46.50–90.50)	67.00 (59.00–75.00)	0.961
LDH, [IU/L]	231.50 (185.50–328.00)	188.00 (174.00–210.00)	0.007
BILIRUBIN, [µmol/L]	8.20 (6.06–11.95)	7.69 (6.79–11.5)	0.771
NH_3_, [µmol/L]	34.55 (25.60–45.85)	32.80 (22.10–40.30)	0.501
INR	1.02 (0.96–1.23)	0.96 (0.91–1.00)	0.003
PT [s]	10.95 (10.40–12.45)	10.30 (9.75–26.30)	0.125
DD, [mg/L]	1.49 (0.77–4.22)	0.30 (0.22–0.35)	<0.001
Na, [mmol/L]	138.00 (136.00–140.00)	140.00 (138.50–141.00)	0.009
K, [mmol/L]	4.27 (3.94–4.64)	4.33 (4.21–4.58)	0.279
Urea, [mmol/L]	5.9 (4.32–7.94)	4.64 (3.79–5.45)	0.020
Lactate, [mmol/L]	1.25 (0.85–1.70)	1.20 (0.80–1.30)	0.419
CK, [U/L]	67.00 (44.50–137.50)	76.00 (61.00–95.50)	0.857
Troponin I hs, [ng/L]	6.06 (2.50–26.30)	3.00 (3.00–3.39)	0.020
Mioglobin, [µmol/L]	56.30 (34.85–105.20)	37.00 (21.70–44.60)	0.042
NT-proBNP, [pg/mL]	196.00 (97.50–695.50)	57.95 (47.15–77.90)	<0.001
Fe, [mmol/L]	5.61 (3.63–12.10)	19.80 (18.20–21.10)	<0.001
Lipase, [U/L]	26.00 (18.00–38.50)	35.00 (25.00–42.50)	0.096
Amylase, [U/L]	48.00 (38.50–73.00)	54.00 (45.00–71.00)	0.731
Cholesterol, [mmol/L]	3.20 (2.65–4.25)	4.60 (4.05–5.40)	<0.001
HDL, [mmol/L]	0.96 (0.74–1.15)	1.43 (1.17–1.62)	<0.001
LDL, [mmol/L]	1.45 (1.10–2.40)	2.50 (2.05–3.00)	0.001
TG, [mmol/L]	1.17 (0.89–1.60)	1.07 (0.85–1.77)	0.917
Protein, [g/L]	61.00 (54.50–65.70)	72.10 (66.90–73.60)	<0.001
Albumin, [g/L]	34.40 (29.70–39.75)	46.45 (43.80–48.45)	<0.001

ALP—alkaline phosphatase, BMI—body mass index, CK—creatine kinase, CRP—C-reactive protein, DD—D dimer, HCT—hematocrit, HDL—high-density lipoprotein, HGB—hemoglobin, INR—international normalized ratio, LDH—lactate dehydrogenase, LDL—low-density lipoprotein, LYM—lymphocytes, NEU—neutrophil, NT-proBNP—N terminal pro b type natriuretic peptide, PCT—procalcitonin, PLT—platelet, PT—prothrombin time, RBC—red blood cell, TG—triglycerides, WBC—white blood cell.

**Table 2:** Comparison between laboratory data collected on 1st and 7th day of hospitalization and control group.

Medians (Q1–Q3)	COVID-19	Control Group	*p*-Value
ALT 1, [U/I]	22.5 (16–53)	18 (15.5–27)	0.199
ALT 2, [U/I]	31.15 (16.7–59.75)		0.049
Difference between serum activity Δ(t2 − t1)	0.712		
AST 1, [U/I]	33 (22–51)	22 (19.5–28)	0.015
AST 2, [U/I]	44.05 (28.95–57.1)		<0.001
Difference between serum activity Δ(t2 − t1)	0.142		
GGTP 1, [U/I]	32 (18.5–82)	18 (14.5–26.5)	0.014
GGTP 2, [U/I]	49.5 (26.35–82.85)		<0.001
Difference between serum activity Δ(t2 − t1)	0.158		
Creatinine 1, [umol/L]	67.85 (57.3–89.3)	71.85 (64.15–83.6)	0.462
Creatinine 2, [umol/L]	69.1 (54.2–86.15)		0.286
Difference between serum levels Δ(t2 − t1)	0.347		
Glucose 1, [mmol/L]	5.78 (4.9–6.96)	5.12 (4.78–5.38)	0.017
Glucose 2, [mmol/L]	4.88 (4.2–6.14)		0.622
Difference between serum levels Δ(t2 − t1)	0.005		
Ferritin 1, [ug/L]	446 (225.5–657)	85.5 (55–143.5)	<0.001
Ferritin 2, [ug/L]	411 (219.5–672.5)		<0.001
Difference between serum levels Δ(t2 − t1)	0.906		
IL-6 1, [pg/mL]	30 (16.1–92.14)	1.5 (1.5–2.29)	<0.001
IL-6 2, [pg/mL]	13 (6.04–43.65)		<0.001
Difference between serum levels Δ(t2 − t1)	0.032		

IL-6—Interleukin 6, GGTP—gamma-glutamyl transpeptidase, AST–aspartate transaminase, ALT–Alanine transaminase.

**Table 3:** Comparison of leptin and visfatin serum levels on 1st and 7th day of hospitalization and control group.

Medians (Q1–Q3)	COVID-19	Control Group	*p*-Value
Leptin 1	7476.3 (2471.6–21,695.15) pg/mL	12,586.75 (4786.33–22,446.51)	0.270
Leptin 2	6543.75 (94,302.5–12,064) pg/mL		0.129
Difference between leptin serum levels Δ(t2 − t1)	−1530.5 (−12,121.3; 3524.03) pg/mL		0.076
Visfatin 1	4.29 (3.0–6.88) ng/mL	37.16 (24.74–50.12) ng/mL	<0.001
Visfatin 2	52.05 (31.2–69.66) ng/mL		0.048
Difference between visfatin serum levels Δ(t2 − t1)Δ(2–1)	47.83 (26.9–62.65) ng/mL		<0.001

**Table 4:** Results of multivariate regression model indicating that COVID-19 is independently lowering visfatin levels at timepoint 1. Corr. R^2^ = 42.22%, *p* < 0.001.

	*p* = Value
COVID-19	0.000000
Age	0.307580
Females	0.071758
Waist circumference	0.054454
BMI	0.093799

## Data Availability

The data that support the findings of this study are available from the corresponding author, upon reasonable request.
